# Functional Redundancy of Septin Homologs in Dendritic Branching

**DOI:** 10.3389/fcell.2017.00011

**Published:** 2017-02-20

**Authors:** Charlotte Kaplan, Mayra Steinmann, Natalia A. Zapiorkowska, Helge Ewers

**Affiliations:** ^1^Department of Biology, Institute of Biochemistry, University of ZurichZurich, Switzerland; ^2^Laboratory of Physical Chemistry, University of ZurichZurich, Switzerland

**Keywords:** septin, dendritic spine, neuron

## Abstract

Septins are cytoskeletal GTPases present in nonpolar heteromeric complexes that assemble in a palindromic fashion from two to eight subunits. Mammalian septins function in several fundamental cellular processes at the membrane-cytoskeleton interface including dendritic branching in neurons. Sequence homology divides the 13 mammalian septin genes into four homology groups. Experimental findings suggest that septin function is redundant among septins from one homology group. This is best understood for the isoforms of the SEPT2 group, which form a homodimer at the center of septin complexes. *In vitro*, all SEPT2-group septins form recombinant hexameric complexes with two copies of SEPT6 and SEPT7. However, it remains unclear to what extent homologs septins can substitute for each other in specific cellular processes. Here, we use the experimental paradigm of dendritic branching in hippocampal rat neurons to ask, to what extent septins of the SEPT2-group are functionally redundant. Dendritic branching is significantly reduced when SEPT5 is downregulated. In neurons expressing SEPT5-shRNA, simultaneously expressed SEPT2-GFP, and SEPT4-GFP colocalize with SEPT7 at dendritic spine necks and rescue dendritic branching. In contrast, SEPT1-GFP is diffusely distributed in the cytoplasm in SEPT5 downregulated neurons and cannot rescue dendritic branching. Our findings provide a basis for the study of septin-specific functions in cells.

## Introduction

The septins are a family of conserved GTPases present in all eukaryotes except land plants and have first been described as cell division cycle (cdc) mutants in yeast (Hartwell, 1971). Mammals express at least 13 different septin genes and differential splicing yields even more polypeptides. Sequence homology analysis classifies septins into four groups: the septin2 group (SEPT1, 2, 4, and 5), the septin3 group (SEPT3, 9, and 12), the septin6 group (SEPT6, 8, 10, 11, and 14; Macara et al., 2002; Pan et al., 2007). SEPT7 forms a homology group in itself and is considered to be essential for the assembly of complexes into filaments (Kinoshita, 2003). Human septins are not expressed at the same levels in all tissues suggesting that individual septins are required for distinct tissue-specific functions (Hall et al., 2005; Peterson and Petty, 2010). However, the function of many septins may be redundant to a certain extent, since knockout mice of SEPT3, 4, 5, and 6 show no detectable or very specific defects (Peng et al., 2002; Ono et al., 2005; Fujishima et al., 2007; Tsang et al., 2008). The knockout of certain septin genes induced an upregulation in protein expression levels of others in the same homology group. These findings suggest a possible functional redundancy between septin genes (Peng et al., 2002). SEPT7 and SEPT9 on the other hand seem to be essential since the respective knockouts are embryonic lethal in mice (Füchtbauer et al., 2011; Menon et al., 2014).

The crystal structure of the heterohexameric human septin complex consists of two SEPT2, SEPT6, and SEPT7 molecules, respectively. The septin complex shows a palindromic arrangement of septin molecules with alternating interactions between their GTPase domains and their combined N-and C-termini. In this complex, two SEPT2 subunits (septin2 group) occupy the center of the complex forming a flexible hinge (Sirajuddin et al., 2007). Two SEPT6 subunits (septin6 group) link the SEPT2 dimer to the SEPT7 molecules at both ends of the complex. SEPT3 group septins can bind to SEPT7 and therefore the human septin protofilmanent exists in heterohexamers and heterooctamers (Kim et al., 2011; Sellin et al., 2011, 2014), however no crystal of the heterooctameric complex exists so far.

SEPT7 is essential for the stability of septin complex heterohexamers and heterooctamers (Sellin et al., 2011). The other septins from the same homology group seem to be incorporated into the complex in a modular fashion (Kinoshita, 2003; Sellin et al., 2011, 2014). For instance, *in vitro* co-expression experiments show that all members of the septin2 homology group assemble into complexes with SEPT6 and SEPT7 in SEPTX/6/7 complexes with a stoichiometry of 2:2:2 *in vitro* (with X standing for SEPT1, SEPT2, SEPT4, or SEPT5; Kinoshita, 2003). While for SEPT1 and SEPT2, no knockout animal has been reported, the SEPT4 and SEPT5 knockout mice yielded highly specific phenotypes, such as male sterility for SEPT4 (Ihara et al., 2005) and problems in synapse maturation (Yang et al., 2010) and a complex behavioral phenotype (Suzuki et al., 2009) for SEPT5. Likewise, in the SEPT5 knockout, the expression of SEPT2, another member of the same subgroup, is up-regulated 3-fold at the protein level (Peng et al., 2002). Nevertheless, there is neither direct evidence that individual septins can substitute for other members of the same group in functional complexes in cells nor that septin complex function strictly depends on the exact subunit composition.

Here we asked whether a single septin could be functionally replaced by a different member of the same homology group in dendritic branching of hippocampal neurons. In neurons the SEPT5/SEPT7/SEPT11 complex is required for the development of a complex branched dendritic morphology (Tada et al., 2007; Garcia et al., 2011). This complex localizes to dendritic branch points and contains with SEPT5, SEPT11 and SEPT7 one member of the septin2 homology group, the septin6 group and SEPT7. The other septin2 group homology members, SEPT1, SEPT2 and SEPT4 are absent from this complex (Tsang et al., 2011).

We find that when SEPT5 is downregulated by RNA-interference, simultaneously expressed GFP-tagged SEPT2 or SEPT4 localizes to dendritic branching points and spine necks. Both, SEPT2-GFP and SEPT4-GFP, colocalize with endogenous SEPT7 and exchange at low rates when photobleached, suggesting successful incorporation into septin complexes. Furthermore, reduced dendritic complexity after SEPT5 downregulation is rescued by simultaneous expression of SEPT2-GFP or SEPT4-GFP. In contrast, SEPT1-GFP is unable to rescue dendritic branching in neurons expressing SEPT5 shRNA. In agreement with this finding, SEPT1 does not colocalize with SEPT7 in neuronal septin structures when SEPT5 protein levels are downregulated and both SEPT1-GFP and SEPT7 are absent from dendritic spine necks in such neurons. Instead, SEPT1-GFP is mobile in the cytoplasm.

In conclusion, we show that SEPT2-GFP and SEPT4-GFP localize to and incorporate into neuronal septin complexes and rescue SEPT5 function in neuronal dendritic morphology. In contrast, SEPT1-GFP shows a different phenotype in neurons and cannot rescue the loss of function of SEPT5. Our results provide evidence for a functional difference between individual septin2 group members in hippocampal rat neurons and an experimental model to further investigate isoform-specific functions of septins.

## Results

### SEPT2-GFP is recruited to the dendritic spine septin complex after downregulation of SEPT5 in hippocampal rat neurons

**Figure 1:**
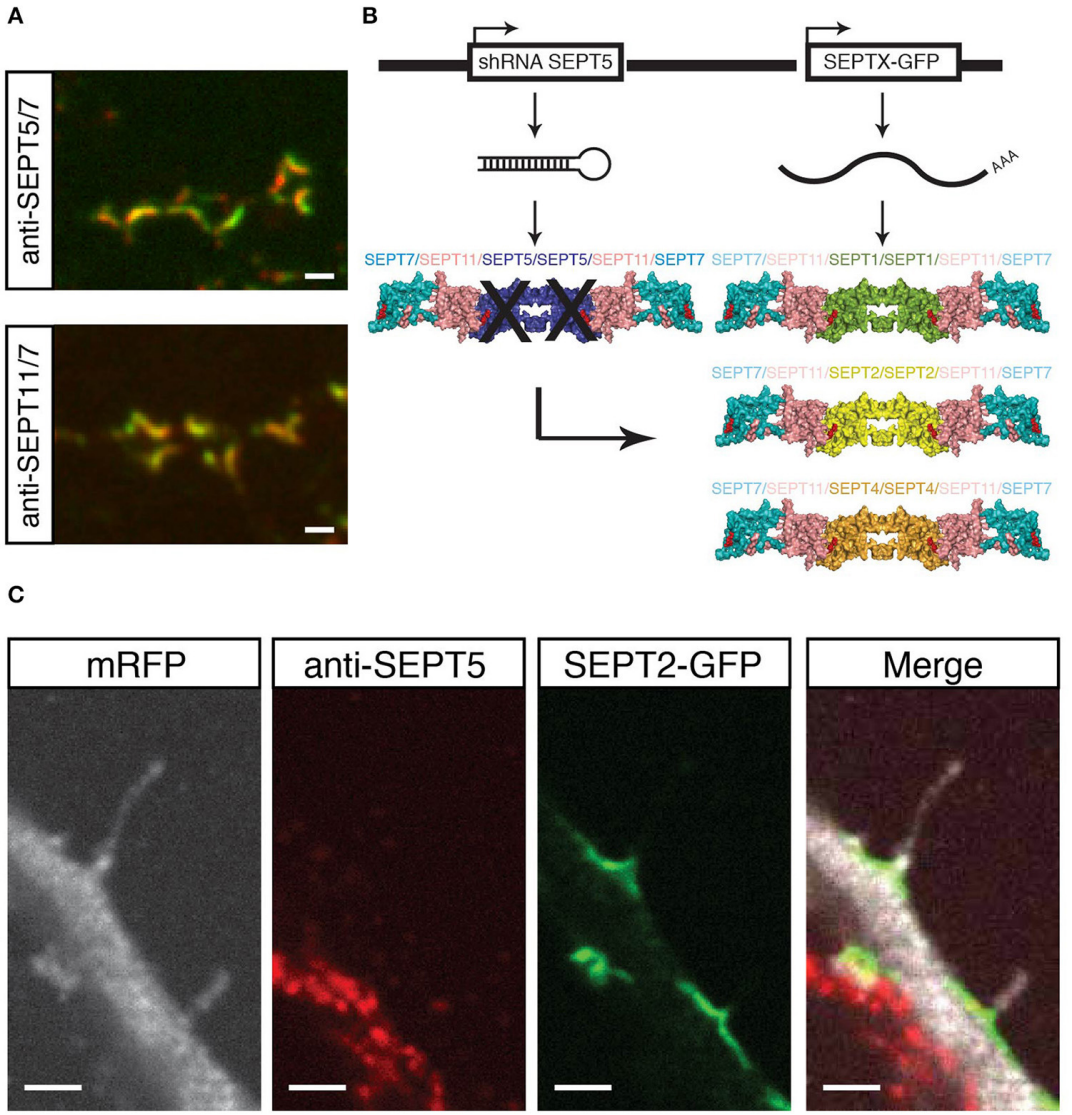
**Experimental setup and proof of principle. (A)** Coimmunostaining of SEPT5 (green) and SEPT7 (red, top) or SEPT11 (green) and SEPT7 (red, bottom) respectively in hippocampal rat neuron cultured *in vitro* and fixed on DIV 21. Scale bar is 1 μm. **(B)** Experimental setup for septin replacement assay. We cotransfected two vectors into cultured hippocampal neurons at DIV 7. One vector bears a shRNA against SEPT5 to knock-down SEPT5 expression, the other expresses a GFP-tagged version of another SEPT2-group septin. The expected results are different recombinant septin complexes in targeted cells. **(C)** Fluorescence micrograph of a neuron cotransfected with a shRNA against SEPT5, a plasmid encoding mRFP as a marker (white) and a plasmid encoding SEPT2-GFP (green). After 5 days of expression, cells were fixed, and immunostained against endogenous SEPT5 (red). Scale bar is 2 μm.

In cultured rat hippocampal neurons, a complex consisting of SEPT5, SEPT7, and SEPT11 has been reported to co-immunoprecipitate and to localize to dendritic branching points (Tada et al., 2007; Garcia et al., 2011). In a first experiment, we confirmed the colocalization of endogenous SEPT5, SEPT7, and SEPT11 in our primary rat hippocampal neuron cultures by immunofluorescence staining (Figure 1A).

We here hypothesized that other members of the septin2 group might replace SEPT5 in its localization and function. The scheme in Figure 1B illustrates our experimental setting on the basis of the crystal structure of the septin complex (Sirajuddin et al., 2007). We aim to downregulate SEPT5 from the functional dendritic spine septin complex by RNA interference. We hypothesized that if we then exogenously expressed individual other septin2 homology group members, SEPT1, SEPT2, and SEPT4 might be able to reconstitute a recombinant complex together with the native SEPT11 and SEPT7 *in vivo*.

To realize this experiment, we cotransfected a vector to knock down SEPT5 by RNA interference with a vector to exogenously express SEPT1, SEPT2, or SEPT4 coupled to GFP, respectively, in hippocampal rat neurons cultured for 7 days *in vitro* (DIV 7). In addition to these two constructs, we cotransfected mRFP as a fluorescent marker to visualize neuronal morphology and neurons were grown for 5 more days to ensure proper downregulation of SEPT5 as done before (Tada et al., 2007; Ewers et al., 2014).

First, we verified the downregulation of SEPT5 by immunostaining the cotransfected neurons against SEPT5 and compared the fluorescence signal intensity to non-transfected neighboring neurons. Cells transfected with shRNA against SEPT5 showed significant reduction in fluorescence intensity levels when compared to control cells (Supplementary Figure 1). We quantified the downregulation by calculating the fluorescence intensity (FI) ratio between FI values measured in transfected over neighboring non-transfected neurons (Supplementary Figure 3A).

As a proof of concept, we assessed the localization of expressed SEPT2-GFP in these cells. SEPT2-GFP localized to the base of dendritic filopodia in an arc-shaped form (Figure 1C), similar to SEPT5, SEPT7, and SEPT11 in control cells. At the same time, the same transfected cell does not show a significant SEPT5 fluorescence signal by immunostaining above background level, while the neighboring cell shows a strong SEPT5 fluorescence signal (Figure 1C). In combination with the quantification of the FI signal this demonstrates that we were able to downregulate SEPT5 in hippocampal rat neurons and to exogenously expressed SEPT2, another member of the septin2 group, simultaneously.

### SEPT2-GFP and SEPT4-GFP, but not SEPT1-GFP localize with endogenous SEPT7 at dendritic spine necks and branches

We next asked, whether generally the exogenous expression of septin2 homology group members in neurons downregulated for SEPT5 resulted in interactions only between the exogenous septins or also with other endogenous septins. To address this question we assessed whether the GFP-fusions of SEPT1, SEPT2, and SEPT4 colocalized with the core member SEPT7 in the arc-shaped structures formed at dendritic spine necks and performed immunofluorescence staining of endogenous SEPT7 in cotransfected neurons.

**Figure 2:**
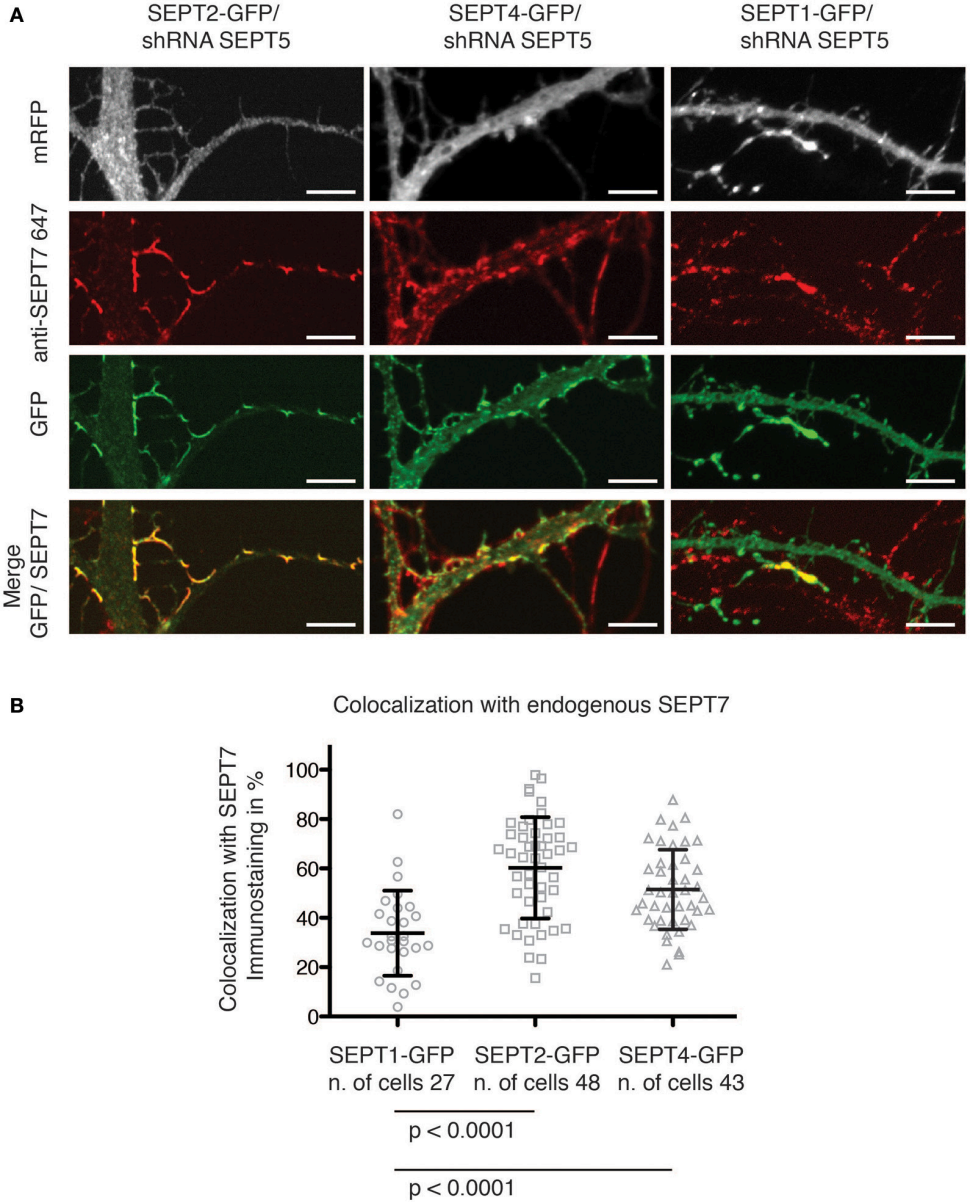
**Colocalization of GFP-tagged SEPT1, SEPT2, and SEPT4 with endogenous SEPT7. (A)** Fluorescence micrographs of dendrites from neurons in which SEPT5 expression was downregulated by shRNA and that express SEPT1-GFP, SEPT2-GFP, and SEPT4-GFP (green) with immunofluorescence staining against endogenous SEPT7 (red). Note the absence of SEPT7 staining from the SEPT1-GFP expressing cell. Scale bars are 1 μm. **(B)** Quantification of the overlap between the fluorescence signals of SEPT7 with SEPT1-GFP (circles), SEPT2-GFP (squares), or SEPT4-GFP (triangles). The percentage of overlap of SEPT1-GFP/SEPT7-AF 647 (*n* = 27 cells) is significantly smaller than that of SEPT2-GFP/SEPT7-AF 647 (*n* = 48 cells, unpaired Mann-Whitney test *p* < 0.0001) and of SEPT4/SEPT7-AF 647 (*n* = 43 cells, unpaired Mann-Whitney test *p* < 0.0001). A Kruskal-Wallis test on the whole dataset resulted in *p* < 0.0001.

We observed that both SEPT2-GFP and SEPT4-GFP localized to the neck of dendritic spines and branching points (Figure 2A). Moreover, a quantitative analysis of the overlap between the septin2 group member's GFP signal and the SEPT7 immunofluorescence signal confirmed that SEPT2-GFP or SEPT4-GFP localize to structures also containing SEPT7 (Figure 2B). In contrast, the SEPT1-GFP fluorescence signal intensity was distributed homogenously over the entire neuron downregulated for SEPT5. Accordingly, we conclude that SEPT1-GFP did not concentrate at dendritic spine necks or branches.

Interestingly, when we quantified the immunofluorescence staining against endogenous SEPT7 in SEPT5-shRNA transfected cells, we observed a significant decrease in the FI levels of SEPT7 in cells depleted of SEPT5 (Supplementary Figure 3B). This is in accordance with published data on SEPT5 knockout mice that showed a clear reduction of SEPT7 protein levels in the brain of these mice (Peng et al., 2002). Remarkably, on the single cell level, we were able to show that expression of SEPT2-GFP and SEPT4-GFP rescues the FI signal of SEPT7 in SEPR5-shRNA treated cells (Supplementary Figures 2, 3B), while the expression of SEPT1-GFP does not.

The septin2 homology group members SEPT1, SEPT2, and SEPT4 are not found in rat neuron septin complexes containing SEPT5 at detectable levels (Tsang et al., 2011). Nevertheless, in our experiments, exogenous expressed SEPT4-GFP as well as SEPT2-GFP localized together with endogenous SEPT7 in higher-order structures at dendritic spine necks and branching points. In addition, SEPT2-GFP and SEPT4-GFP expression remarkably restored SEPT7 fluorescence intensity levels. Our data demonstrate that some but not all septin2 homology group members can replace SEPT5 in neuronal septin complexes.

### SEPT2-GFP and SEPT4-GFP, but not SEPT1-GFP assembles in a stable complex at dendritic spine necks and filopodia

We have shown before by fluorescence recovery after photobleaching (FRAP) that septins form stable, immobile higher-order structures at spine necks (Ewers et al., 2014). To examine whether the septin2 homologs were incorporated in such stable higher-order structures in hippocampal rat neurons after SEPT5 knockdown, we performed FRAP experiments of overexpressed SEPTX-GFP fusion proteins at dendritic spine necks.

**Figure 3:**
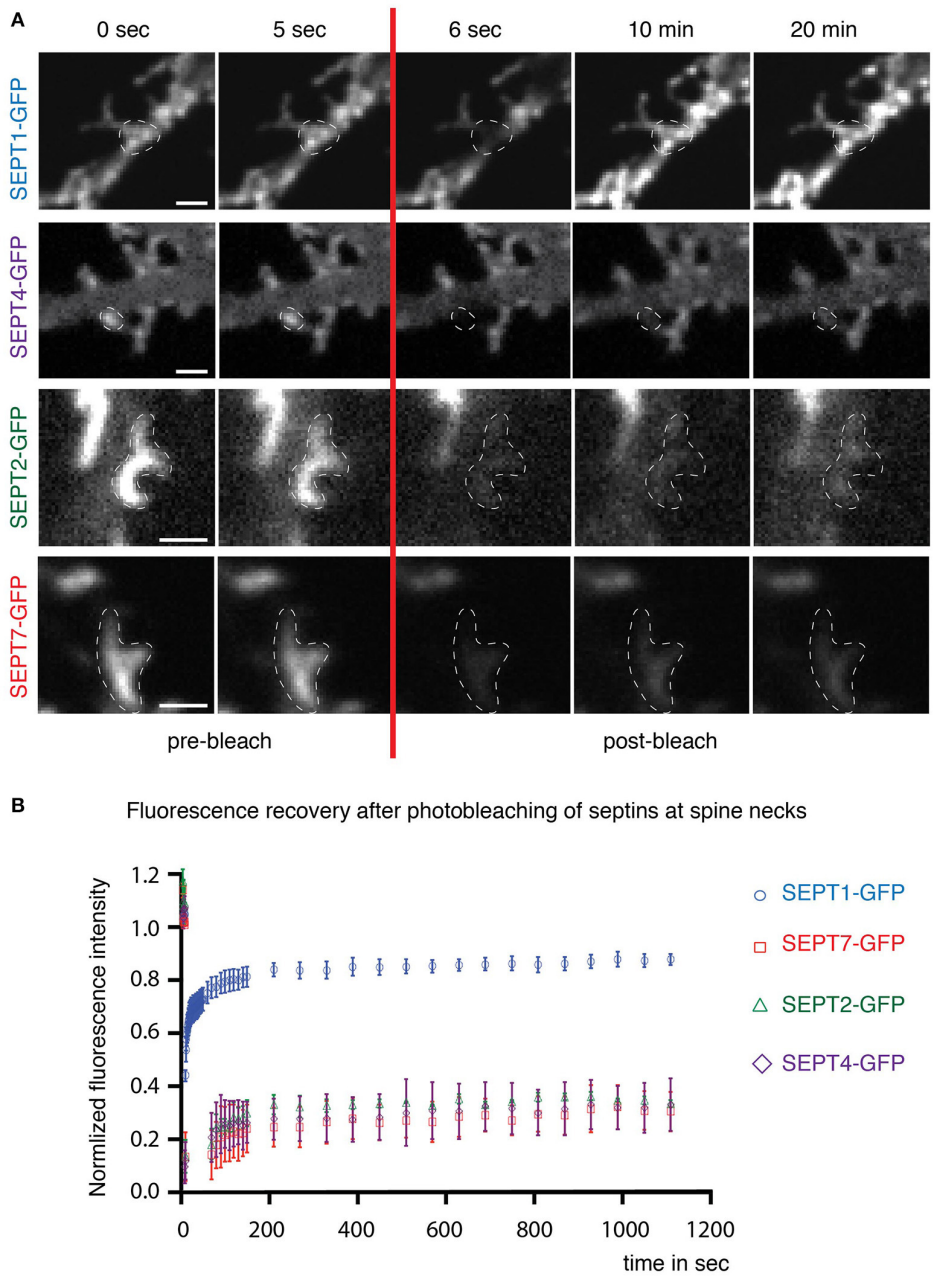
**Dynamics of septin2 group homologs and SEPT7 at dendritic spine necks. (A)** Fluorescence recovery after photobleaching (FRAP) time course experiment on septin structures at the base of dendritic spines. Two images before (0 s; 5 s) and three after (6 s; 10 min; 20 min) the bleaching pulse are shown. A broken white line emphasizes the photobleached and quantified area. Note the homogenous distribution of SEPT1-GFP and the discrete arc-shaped structures at the base of spines formed by SEPT2-GFP, SEPT4-GFP, and SEPT7-GFP. Scale bars are 1 μm. **(B)** Quantification of FRAP experiments performed in live cells at DIV 7 + 7 to DIV 7 + 9. The recovery of the fluorescence signal is plotted against the time using the mean value and the standard error of the mean from three independent experiments. Total number of septin structures from all three experiments: SEPT2-GFP, green diamonds: *n* = 30, SEPT1-GFP, blue circles: *n* = 33, SEPT4-GFP, purple triangles: *n* = 33, SEPT7-GFP, red squares: *n* = 34.

SEPT1-GFP fluorescence (Figure 3A, first row) recovered quickly at dendritic spine necks (Figure 3B) with a mobile fraction of about 80%, consistent with a cytoplasmic localization. In contrast, only a small fraction of SEPT2-GFP and SEPT4-GFP in SEPT5 downregulated neurons exchanged slowly between the septin higher-order structures located at the base of spines or filopodia in hippocampal rat neurons and the soluble pool in the cytoplasm (Figure 3A, second and third row and Figure 3B). This slow recovery agrees with the behavior of SEPT7-GFP in neurons with endogenous SEPT5 expression levels (Figure 3A, fourth row and Figure 3B). Consequently, we conclude that SEPT2-GFP, SEPT4-GFP, and SEPT7-GFP are stably integrated into the septin structure at the base of dendritic spines.

### SEPT2-GFP and SEPT4-GFP, but not SEPT1-GFP rescues dendritic branching in hippocampal rat neurons

**Figure 4:**
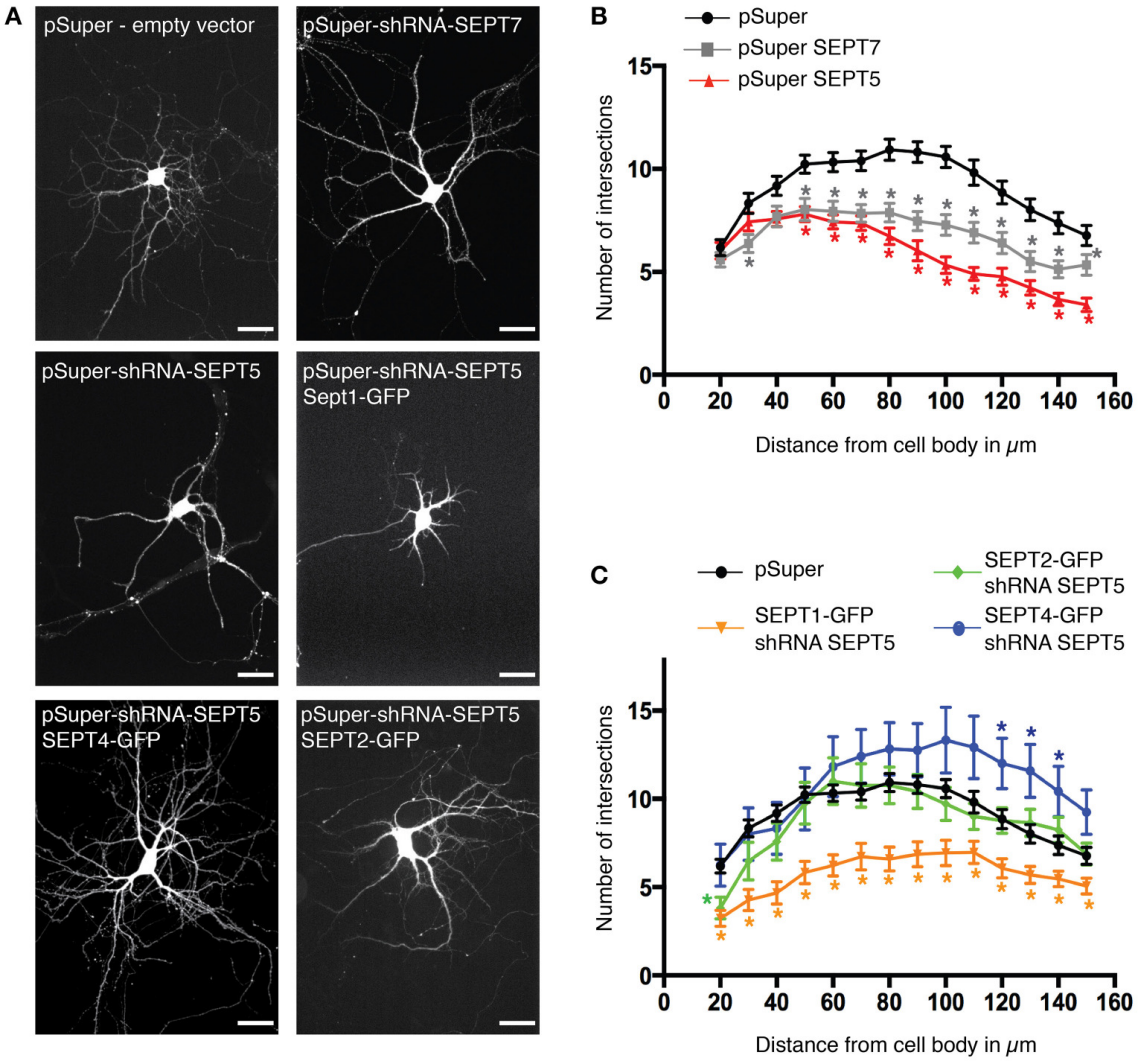
**Rescue of SEPT5 function in dendritic branching. (A)** Fluorescence micrographs of hippocampal neurons coexpressing mRFP as a marker for cell morphology with shRNA constructs and constructs expressing GFP-tagged septins. Shown is the mRFP channel in grayscale to visualize the cell outline. Scale bars are 50 μm. **(B)** Quantification of dendritic branching by Sholl analysis in control cells and after shRNA-mediated knockdown of SEPT5 or SEPT7 expression. The branching complexity is significantly reduced in neurons transfected with shRNA against SEPT5 (*n* = 30 cells, red triangle) or SEPT7 (*n* = 32 cells, gray square) when compared to control cells (*n* = 43 cells, black circle). Significance difference between the control cells and the knock down of SEPT5 or SEPT7 is highlighted by an asterisk next to the data point in the respective color code when the *p* < 0.02 (*p*-values from a multiple *t*-test in Supplementary Table 2). **(C)** Quantification of dendritic branching by Sholl analysis in control cells and after shRNA-mediated knockdown of SEPT5 expression with simultaneous expression of SEPT1-GFP (*n* = 30 cells, yellow triangles), Sept2-GFP (*n* = 27 cells, green diamonds), or SEPT4-GFP (*n* = 12 cells, blue circles). Significance difference between the control cells and the three different conditions of SEPT5 knock down and simultaneous expression of another SEPT2 group member is highlighted by an asterisk next to the data point in the respective color code when the *p* < 0.02 (*p*-values from a multiple *t*-test are listed in Supplementary Table 2).

A functional septin complex is required for proper dendritic branching in hippocampal neurons (Tada et al., 2007) since the knockdown of either SEPT5 or SEPT7 leads to reduced dendritic arbor complexity. Simultaneously, the downregulation of either SEPT5 or SEPT7 also caused a reduction in the expression level of the respective other. We thus hypothesized, that SEPT1-GFP, which is unable to rescue septin complex localization, might not be able to rescue the dendritic branching defect caused by SEPT5 downregulation, while SEPT2-GFP and SEPT4-GFP might. To test this hypothesis, we cotransfected either SEPT1-GFP, SEPT2-GFP, or SEPT4-GFP with shRNA against SEPT5 and mRFP as a tracer at DIV7 and fixed the neurons after 5 days of combined SEPT5 knockdown and SEPTX-GFP expression. Images of the entire neuron including its dendrites were acquired and the complexity of dendrite branching was analyzed by Sholl analysis (Sholl, 1953). Consistent with previous results, dendritic complexity was strongly reduced in neurons downregulated for SEPT7 and SEPT5 (Figures 4A,B, *p*-values in Supplementary Table 2). Exogenous expression of either SEPT4-GFP or SEPT2-GFP in these cells rescued the dendritic branching morphology (Figures 4A,C, *p*-values in Supplementary Table 2). In line with the FRAP results and colocalization experiments, SEPT1-GFP on the other hand was not able to restore dendritic branching complexity in cells treated with shRNA against SEPT5. We conclude that the function of SEPT5 in dendritic branching is not redundant between all septin2-group members and can be rescued by SEPT2 and SEPT4 only.

## Discussion

Septin complexes are assembled from several different septins from the four groups classified by sequence homology in dependence on their tissue-specific expression. In this study, we investigated the hypothesis that septins belonging to the same homology group can substitute for each other in the septin complex and in cellular function.

Several *in vitro* and *in vivo* findings are supportive of this notion. Recombinant expression of SEPT6 and SEPT7 together with members of the septin2 homology group in insect cells showed that each member of this homology group can assemble into a complex with SEPT6 and SEPT7 (Kinoshita, 2003). Furthermore, the relatively mild phenotypes of the knock-out of septin2 group members SEPT4 and SEPT5 in mice suggest a functional redundancy of homologs within the same septin group. For instance, in knock-out mice of SEPT5, SEPT2 from the same homology group showed increased expression levels (Peng et al., 2002). However, evidence for a functional redundancy of septins from the same homology group on the cellular level is lacking. Hence, we choose to investigate whether other homologs from group 2 can substitute for SEPT5 in its specific function in dendritic branching. In hippocampal neurons, SEPT5 colocalizes at dendritic branching points and at the neck of spines with SEPT11 and SEPT7 (Tada et al., 2007; Garcia et al., 2011). SEPT5 knockdown by shRNA reduces the complexity of the dendritic arbor (Tada et al., 2007; Garcia et al., 2011). SEPT5 is the only member exclusively expressed at high levels in hippocampal rat neurons (Tsang et al., 2011), rendering it an optimal system to study septin-specific function. In our experimental set up, we thus downregulated SEPT5 by shRNA and simultaneously expressed septin2 group members SEPT1-GFP, SEPT2-GFP, or SEPT4-GFP to investigate, whether they would rescue SEPT5 function in dendritic branching.

When we exogenously expressed SEPT2-GFP and SEPT4-GFP in SEPT5 downregulated neurons, both colocalized with endogenous SEPT7 at dendritic branching points and dendritic spine necks. In contrast, SEPT1-GFP showed no significant colocalization with SEPT7. The integration of exogenously expressed septins into native complexes has been demonstrated before. In K562 cells, the expression of exogenous septins close to the endogenous level leads to their incorporation into septin complexes and eventually to replacement of the endogenous version in these (Sellin et al., 2011). We chemically fixed the neurons after 5 days of knockdown of endogenous SEPT5 and overexpression of another group 2 homology member. At this time, exogenous SEPT2-GFP and SEPT4-GFP both were found in discrete localizations where they quantitatively colocalized with endogenous SEPT7 and endogenous SEPT7 colocalized quantitatively with them. We thus conclude that SEPT2-GFP and SEPT4-GFP are incorporated into endogenous septin complexes.

In the same study, Sellin and coworkers showed when the core unit consisting of SEPT2 and/or SEPT5 was depleted in K562 cells they found a significant decrease in heteromer assembly of SEPT7, SEPT9, and septin6 group members by ultracentrifugation analysis. This finding suggests that the core unit of septin2 group members is important to nucleate septin complex assembly. In our study we did not detect a strong colocalization of SEPT1-GFP with SEPT7 and SEPT1-GFP did not localize with SEPT7 to dendritic spine necks and branching points. In addition, immunolabeling of SEPT7 in neurons in which SEPT1-GFP substitutes SEPT5 showed a decrease in the intensity of the SEPT7 fluorescence signal. This suggests that SEPT1-GFP was not able to nucleate septin complex formation in hippocampal rat neurons, whereas SEPT2-GFP and SEPT4-GFP were able to induce proper complex formation and as a result, proper localization.

SEPT7 stability in cells requires the presence of septin6 and septin2 group homology members and vice versa. SEPT7 by itself is insoluble when expressed recombinantly in bacteria or eukaryotic cells (Kinoshita, 2003). At the same time, SEPT7 binding to SEPT2 and SEPT6 containing heteromers contributes to their stability. Hence, we can conclude that SEPT7 colocalization with SEPT2-GFP and SEPT4-GFP results from productive complex assembly. In addition, the GFP-tag seemingly does not interfere with the function of the septin2 core members to initiate septin complex assembly. Taken together it seems that the cause of the failure of SEPT1-GFP to rescue dendritic branching lies in its failure to recruit functional septin complexes to dendritic branching points and possibly even failure to incorporate into functional septin complexes. This stands in contrast to biochemical experiments demonstrating assembly of SEPT1/SEPT6/SEPT7 complexes and thus that SEPT1 homodimers can exist at the core of a stable septin complex. In neuronal complexes, SEPT11 is prominent as a SEPT6 group member linking the septin2 group members and SEPT7. A failure of SEPT1-GFP to form functional complexes could thus be due to problems with the interaction of the SEPT1 GTPase interface with the septin6 group member SEPT11 in neuronal septin complexes. A biochemical interactome study of septins found all septin2 group members except SEPT2 to interact with SEPT11 (Nakahira et al., 2010), but detailed biochemical studies involving SEPT1 are lacking.

On the other hand, it is clear that septin2 group members bear different posttranslational modifications. The serine/threonine kinase Aurora-B for example phosphorylates SEPT1 *in vitro* and colocalizes with SEPT1 at the midbody during cytokinesis (Qi et al., 2005). SEPT1 knock-down in mouse oocytes causes spindle defects and impaired chromosome congression as well (Zhu et al., 2011) suggesting a functional connection between SEPT1 and AuroraB. SEPT1 contains three serines (Ser248, Ser307, and Ser315) phosphorylated by AuroraB (Qi et al., 2005) that in other members of the septin2 homology group are missing. While in non-mitotic hippocampal neurons, this very mechanism will likely not play a role, unknown regulatory mechanisms based on posttranslational modifications may allow SEPT2-GFP and SEPT4-GFP to form complexes with SEPT11 and SEPT7 at dendritic spine necks, but not SEPT1-GFP.

In our study, we used the longest available splice isoforms of SEPT1, SEPT2, and SEPT4, respectively (Supplementary Figure 4). While in rats only one SEPT2 peptide is expressed, for other SEPT2 group members, especially SEPT4, multiple known and predicted splice isoforms exist (Supplementary Table 3). Our conclusions are thus limited to the specific isoforms of each homolog that we expressed in the SEPT5 downregulated neurons. These isoforms contain all canonical septin features, such as the N-terminal domain with the polybasic region, the GTPase domain, the septin unique element and the C-terminus with the coiled-coil domain (Supplementary Figure 4) and very little difference in their amino acid sequence length. All the more this suggests that the functional difference between the SEPT1 isoform and SEPT5, SEPT2, and SEPT4 isoforms used here, lies rather not in specific domains, but on the level of the specific amino acid sequence of different isoforms.

Our data suggest that significant basic functions can be redundant between septin2 group homologues. SEPT2 and the longest isoform of SEPT4, respectively, instead of SEPT5, rescued a complex cellular process such as dendritic branching. In this particular function, some septins may rather play a structural than a functional role, which would be executed on the level of the complex or by a different septin within it instead. SEPT1 could not rescue dendritic arborization and could not rescue SEPT7 expression. This may be due to a requirement of a specific SEPT6 group septin as a “bridge” to SEPT7 to form complexes that is not expressed in neurons. It may also point to a function of SEPT1 outside of the septin complex.

Septin biology with 13 genes in mammals and many more splice isoforms that assemble in a modular fashion into functional complexes is very difficult to tackle experimentally. The discovery and investigation of isoform specific functions using established experimental paradigms promises to be an important tool to further our understanding of cellular functions of septins in health and disease.

## Materials and methods

### Cloning of septin constructs

The plasmids pSuper-control (scrambled shRNA), pSuper-Sept5, pSuper-Sept7, pßactin-Sept7-GFP, and pßactin-Sept2-GFP were kind gifts from the laboratory of Morgan Sheng. The cloning as well as the design of the shRNA are described in Tada et al. (2007). The cDNAs encoding for *Rattus norvegicus* SEPT1 and SEPT4 originate from the mammalian gene collection (SEPT1: MGC109124; SEPT4: MGC156532). Further detailed information about the SEPT2 group isoforms used in this study is listed in Supplementary Table 3. The plasmids used in this publication were generated by inserting Sept1 or Sept4 into pßactin-GFP using BsrgI and ClaI sites. All plasmids used in this study are listed in Supplementary Table 1.

### Hippocampal culture, transfection, and immunocytochemistry

Humane killing for preparation of rat primary hippocampal neurons conformed to local King's College London ethical approval under the UK Supplementary Code of Practice, The Humane Killing of Animals under Schedule 1 to the Animals (Scientific Procedures) Act 1986, or in accordance with the guidelines issued by the Swiss Federal Act on Animal Protection, respectively. All efforts were made to minimize animal suffering and to reduce the number of animals used. The preparation and cultivation of primary hippocampal neurons from E18 Sprague-Dawley rats was performed as described previously (Kaech and Banker, 2006): rat embryos removed from a time-mated sacrificed rat were placed in ice-cold HBBS and decapitated. Brains were transferred into a new dish of ice-cold HBSS, cerebral hemispheres were removed and freed from their meninges. The hippocampi were dissected and collected in ice-cold HBSS. Hippocampi were then incubated in trypsin solution (Life Technologies) prewarmed to 37°C for 15 min. Singularization of neurons was performed by carefully pipetting up and down in a 1000 μl pipette tip. Neurons were plated on polylysine pre-coated coverslips with a density of 2–3 × 10^5^ cells/dish and cultured in neurobasal medium supplemented with B-27 and GlutaMax (all from Life-Technologies) at 37°C in 5% CO_2_. After 3 days *in vitro* (DIV) Cytarabine was added with a concentration of 5 μM to inhibit cell growth.

Depending on their developmental stage neurons were transfected using Lipofectamin 2000 (Life Technologies, cat. no. 11668-027) between DIV 7 or DIV 8. For the control experiment and septin downregulation the following amounts of DNA were transfected per 6 cm dish: pSuper-control: 0.5 μg, pSuper-SEPT5: 0.5 μg, pSuper-SEPT7: 0.9 μg; mRFP was transfected as a marker to identify transfected cells in the same respective amounts. The concentration of the simultaneously cotransfected SEPTX-GFP constructs (0.4–0.8 μg plasmid) was adjusted such that no overexpression artifacts were observed and localization profiles looked similar to that of endogenous septins.

Transfected neurons were cultivated further for at least 5 days. Fixation was performed for 10 min at room temperature in 4% paraformaldehyde and 2% sucrose in PBS (pH 7.4). Cells were washed for 15 min in PBS containing 50 mM NH_4_Cl and treated for 5 min with 0.25% Triton X-100. Subsequently, cells were blocked with 4% goat serum, 1% bovine serum albumin (BSA) and 0.002% NaN_3_ in PBS for 45 min. Primary antibody treatment was done for 2 h at room temperature (RT) or overnight at 4°C using either rabbit anti-SEPT7 (1:1000, kind gift from M. Kinoshita, Nagoya University, Japan), guinea-pig anti SEPT7 (Tada et al., 2007), rabbit anti-SEPT5 (1:200, kind gift from Bill Trimble, University of Toronto, Canada) or rabbit anti-SEPT11 (kind gift from Barbara Zieger, University of Freiburg). Secondary antibody staining was performed with anti-rabbit AlexaFluor647 and anti-guinea-pig AlexaFluor568 (Life Technologies) for 30 min at RT. Cells were washed twice in 1% BSA and 0.002% NaN_3_ in PBS and mounted on Vectashield H-1000 (Vector Laboratories, Inc. CA, USA).

### Image acquisition and quantification

Confocal images for the analysis of fluorescence intensity, colocalization or morphology were acquired on a custom-made spinning disk confocal fluorescence microscope based on an Olympus IX71 (Zeiss) equipped with a CoolSnap HQ2 CCD-camera (Photometrics) and controlled by the MAG Biosystems acquisition software MetaMorph, V.7.6.4.0. Objectives with a 20x (Olympus UPlanSApo NA 0.75 air) or 40x (Olympus PlanSApo N, NA 1.42 oil) magnification were used. Z-stacks were acquired through the entire cell body when cells were imaged with the 40x objective. For further analysis the z-stacks were transformed into maximum intensity projections using NIH ImageJ.

### Fluorescence intensity measurements

Efficient down-regulation of SEPT5 and SEPT7 in neurons transfected with shRNA was quantified using immunofluorescence images of transfected cells compared to non-transfected cells in the same scan. The mean fluorescence intensities were measured on maximum projections for three different regions of interest (ROI) in branched dendrites of transfected and neighboring non-transfected neurons. Next to the dendrites the background fluorescence was determined and subtracted from the measured dendritic regions. A ratio from these obtained values of transfected cells to non-transfected cells was calculated: a ratio close to 1 means no change in the protein expression level, whereas a ratio <1 means a decreased protein expression level caused by RNA interference. Statistical analysis was performed using Prism Version 6 (Graph Pad software).

### Colocalization analysis of septins in fixed neurons

Colocalization analysis was performed in NIH ImageJ. To do so, the individual fluorescence channels were converted into black (0) and white (1) pixel binary images. The two binary images were then multiplied. To determine the percentage of the overlapping fluorescence signal the number of white pixels in the multiplied image was divided by the number of white pixels in the image of the overexpressed septin of interest. Statistical analysis was performed using Prism Version 6 (Graph Pad software).

### Morphological analysis of fixed neurons

Sholl analysis was performed using the Sholl Analysis Plugin v1.0 of NIH ImageJ. To avoid false crossings due to background fluorescence resulting from other neurons the images were first processed with the Neurite Tracer of the NIH ImageJ plugin NeuronGrowth (Fanti et al., 2008). The Sholl Analysis plugin itself was not able to detect all dendrites due to variations in fluorescence intensity along individual dendrites. Therefore, binary images of the hand-traced dendrites were created with equalized gray values in the NeuronGrowth plugin.

The middle of the cell body was selected manually. The other parameters in the Sholl Analysis plugin were set such that a concentric circle starting at a radius of 20 μm increased in steps of 10 μm and ended at a radius of 150 μm (radius span = 0 μm, span type = mean). The algorithm counted how many intersections occurred between the concentric circles and the dendrites to determine the dendrites' morphological complexity. Statistical analysis was performed using Prism Version 6 (Graph Pad software).

### Live cell imaging and fluorescence recovery after photobleaching (FRAP) experiments

Live-cell FRAP measurements were performed using the custom-made spinning disk confocal microscope as described under “Image Acquisition And Quantification.” The 100x Olympus (PlanSApo N, NA 1.40 oil) objective and the sample holder stage were heated to a temperature of 37°C. Neurons (DIV7 + 7 to DIV7 + 9) were imaged in a buffer containing 145 mM NaCl, 5 mM KCl, 10 mM Glucose, 10 mM Hepes, 2 mM CaCl_2_, 1 mM MgCl_2_, and 0.2% BSA. The FRAP laser and time course imaging were controlled using I-Las Version 1 of the Mag Biosystems software and images were captured with an evolve EM-CCD camera (Photometrics). Five prebleaching images were recorded at 1 s intervals. A single 100 ms bleaching pulse was applied to various circular regions along the dendrite. Each region had a size of approx. 0.7 μm diameter. Per measurement 6–8 ROI at dendritic spine necks were bleached. The duration of post bleach time course imaging was adjusted to the observed recovery of the fluorescence signal. SEPT2-GFP, SEPT4-GFP, and SEPT7-GFP expressed in SEPT5 downregulated neurons were imaged for 20 min with intervals of 60 s. To record the faster recovery in SEPT1-GFP expressing cells the first 10 images were recorded at 1 s intervals followed by 20 images at 1.5 s intervals, 10 images at 20 s intervals and 20 images at 60 s intervals.

The time course images were corrected for cellular movement and stage drift during the acquisition. The fluorescence intensity of the bleached ROIs, the whole cell and the background were analyzed using NIH ImageJ. The fluorescence intensity signal was double normalized as described earlier (Phair and Misteli, 2000). All data obtained from the images analysis was plotted and statistical analyzed in Prism Version 6 (Graph Pad software).

## Author contributions

HE conceived the project; HE and CK designed experiments; CK, MS, and NZ performed experiments; CK, HE analyzed data; CK prepared the draft of the manuscript and HE. edited it.

### Conflict of interest statement

The authors declare that the research was conducted in the absence of any commercial or financial relationships that could be construed as a potential conflict of interest.

## Supplementary material

Supplementary Figure 1 **Immunofluorescence staining against SEPT5 after treatment of neurons with shRNA against SEPT5**. Immunofluorescence images show example cells transfected with shRNA against SEPT5, mRFP as a marker (shown in white, top row), endogenous SEPT5 detected by immunofluorescence (red, second row), and SEPTX-GFP (green, third row), where X stands for SEPT1, SEPT2, or SEPT4. SEPT2-GFP and SEPT4-GFP signals cluster along the dendrite, whereas SEPT1-GFP localizes homogenously within the cytoplasm (all in green). Scale bars are 2 μm.

Supplementary Figure 2 **Immunofluorescence staining against SEPT7 after treatment of neurons with shRNA against SEPT5**. Immunofluorescence images show example cells transfected with shRNA against SEPT5, mRFP as a marker (shown in white, top row), endogenous SEPT5 detected by immunofluorescence (red, second row), and SEPTX-GFP (green, third row), where X stands for SEPT1, SEPT2, or SEPT4. Antibody immunostaining against SEPT7 (red) reveals a strong overlap between SEPT2-GFP/SEPT7 or SEPT4-GFP/SEPT7 in the merged images. SEPT2-GFP and SEPT4-GFP localize to higher-order structures at the dendritic spine necks, whereas the SEPT1-GFP signal is spread all over the cell (all in green). Scale bars are 2 μm.

Supplementary Figure 3 **Quantification of immunofluorescence data as shown in Supplementary Figures 1, 2. (A)** The boxplot illustrates the fluorescence intensity ratios of neurons immunostained against SEPT5. The transfection of the empty pSuper vector served as a negative control, whereas the same vector containing the shRNA against SEPT5 was employed as a positive control. In all neurons cotransfected with shRNA against SEPT5 and SEPTX-GFP, SEPT5 was efficiently reduced. An unpaired Mann-Whitney test was performed to assess significance (^****^*p* < 0.0001). “*n*” stands for the number of cells. **(B)** The fluorescence intensity ratios of neurons immunostained against SEPT7 illustrate that transfection of shRNA against SEPT7 efficiently downregulates the protein levels of SEPT7 in neurons (^****^*p* < 0.0001). In neurons downregulated for SEPT5 the fluorescence intensity ratio of SEPT7 is reduced slightly but significantly (^**^*p* = 0.0029). A significant increase of the FI SEPT7 signal in SEPT2-GFP/shRNA SEPT5 expressing neurons and SEPT4GFP/shRNA SEPT5 expressing neurons compared to shRNAS5 was detected (^****^*p* < 0.0001). No statistically significant difference was observed for the FI SEPT7 signal from SEPT1-GFP/ shRNA SEPT5 expressing neurons compared to the FI SEPT7 signal from only shRNAS5 expressing neurons (ns, *p* = 0.1221). An unpaired, two-tailed Mann-Whitney test was performed to assess significance. “*n*” stands for the number of cells.

Supplementary Figure 4 **Scheme showing SEPT2 group isoforms of ***Rattus norvegicus*****. All isoforms listed are known sequences from cDNA cloning. Shown are the common sequence features that the SEPT2 group isoforms share: a black bar describes the N-terminus that is very variable in length between the different SEPT2 group isoforms. The polybasic region is shown by a black box. The green and red boxes comprise the GTPase domain and septin unique element. All isoforms have a coiled-coil domain in the C-terminus in common. Isoforms that were used in this study are highlighted with a red square.

Supplementary Table 1 **Plasmids used in this study**.

Supplementary Table 2 *****P***-values of Sholl analysis**. *P*-values were calculated to compare the control morphology with neurons depleted of SEPT7 or SEPT5 and with neurons depleted of SEPT5 and cotransfected with SEPT1-GFP, SEPT2-GFP, or SEPT4-GFP. *P* < 0.02 are highlighted in red.

Supplementary Table 3 **SEPT2 group isoforms of ***Rattus norvegicus*****. We listed all cloned and predicted isoforms with the Uniprot and NCBI Protein ID reference. We provide the literature reference for the isoforms used in this study in the last column.
