# Supplementary figures and images for: Functional Redundancy of Septin Homologs in Dendritic Branching
Source: Front Cell Dev Biol. 2017 Feb 20;5:11. doi: 10.3389/fcell.2017.00011 (PMC5316521; doi:10.3389/fcell.2017.00011)

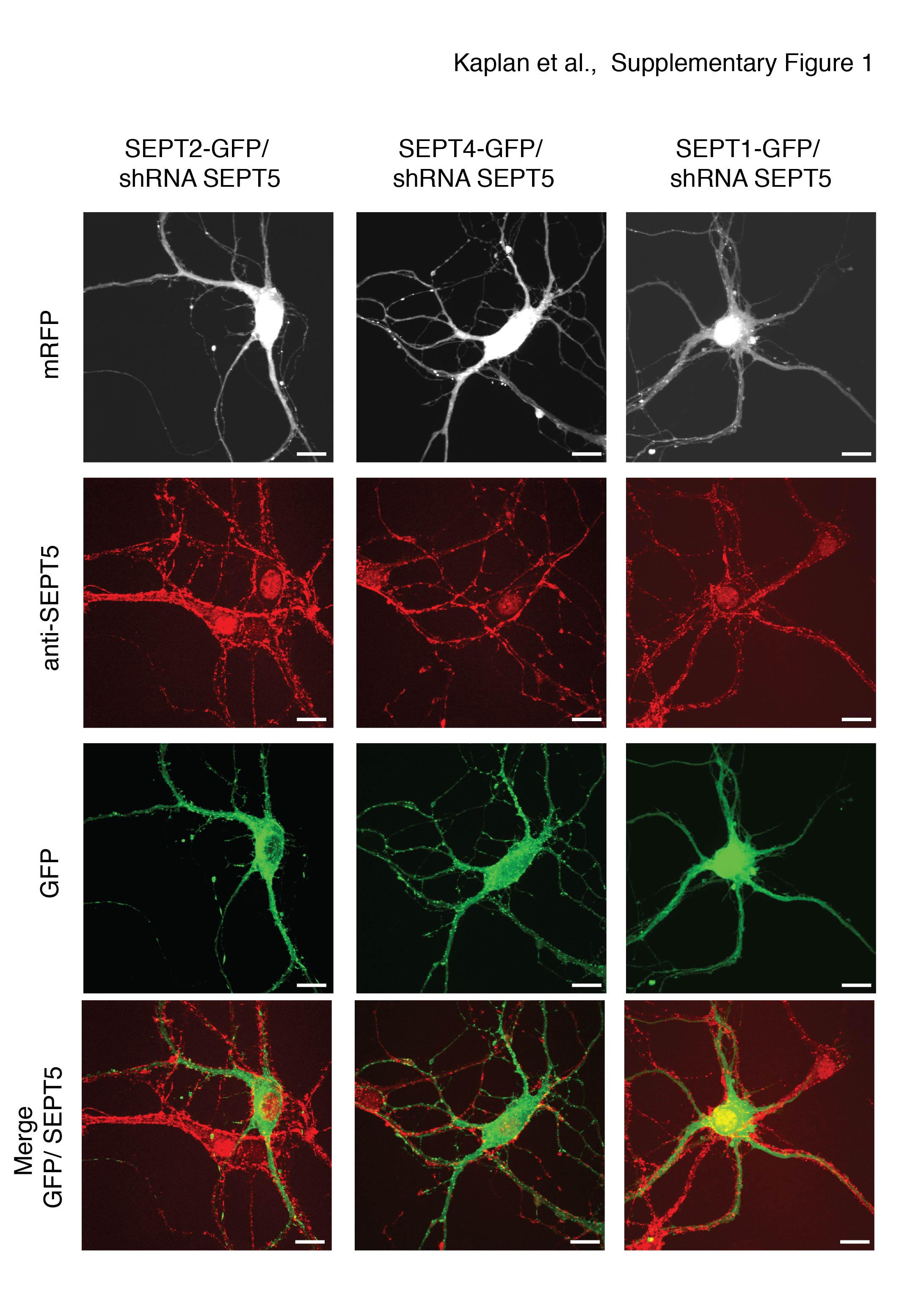

Supplement: Supplementary Figure 1 — Immunofluorescence staining against SEPT5 after treatment of neurons with shRNA against SEPT5. Immunofluorescence images show example cells transfected with shRNA against SEPT5, mRFP as a marker (shown in white, top row), endogenous SEPT5 detected by immunofluorescence (red, second row), and SEPTX-GFP (green, third row), where X stands for SEPT1, SEPT2, or SEPT4. SEPT2-GFP and SEPT4-GFP signals cluster along the dendrite, whereas SEPT1-GFP localizes homogenously within the cytoplasm (all in green). Scale bars are 2 μm. [file Image1.JPEG]

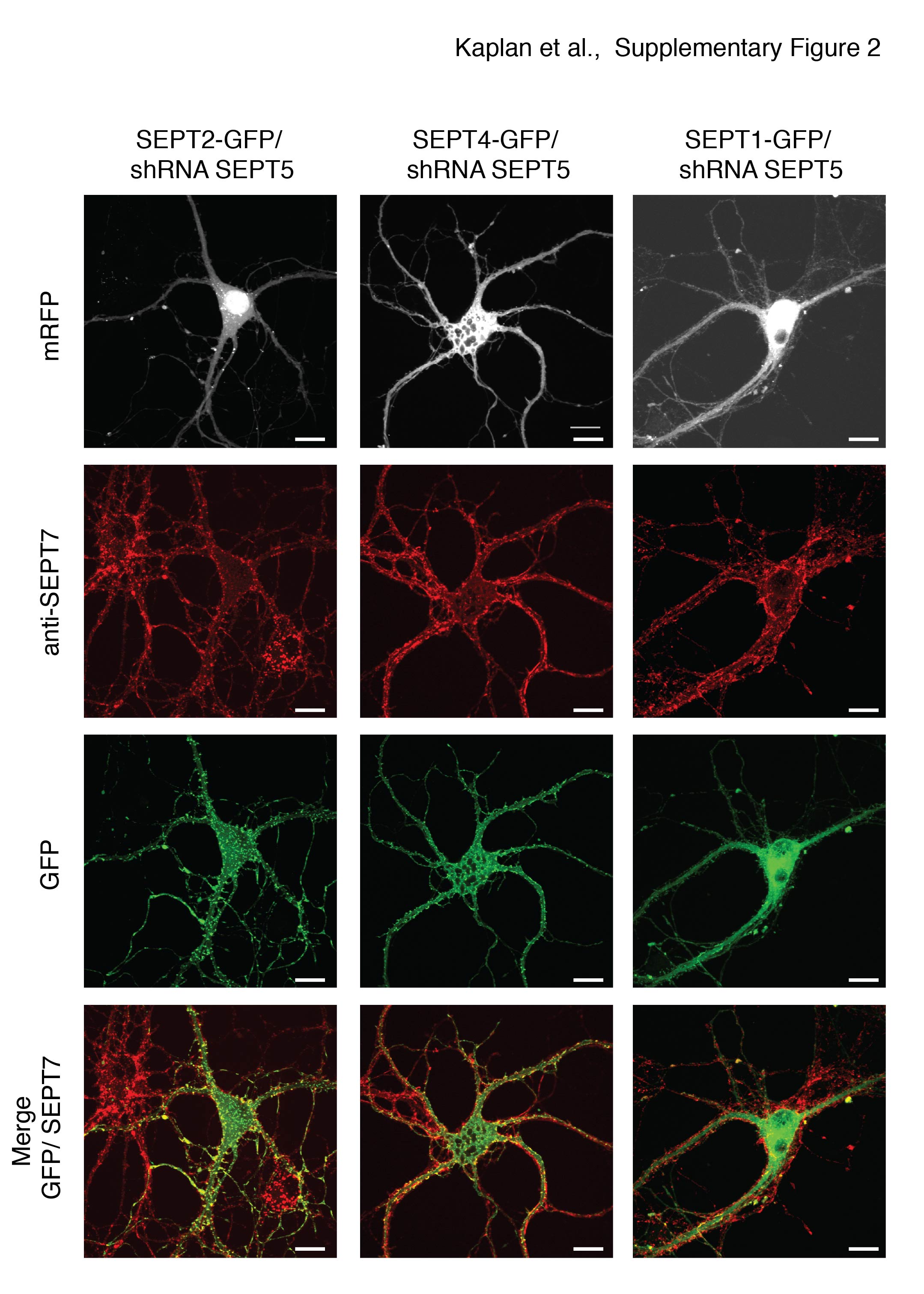

Supplement: Supplementary Figure 2 — Immunofluorescence staining against SEPT7 after treatment of neurons with shRNA against SEPT5. Immunofluorescence images show example cells transfected with shRNA against SEPT5, mRFP as a marker (shown in white, top row), endogenous SEPT5 detected by immunofluorescence (red, second row), and SEPTX-GFP (green, third row), where X stands for SEPT1, SEPT2, or SEPT4. Antibody immunostaining against SEPT7 (red) reveals a strong overlap between SEPT2-GFP/SEPT7 or SEPT4-GFP/SEPT7 in the merged images. SEPT2-GFP and SEPT4-GFP localize to higher-order structures at the dendritic spine necks, whereas the SEPT1-GFP signal is spread all over the cell (all in green). Scale bars are 2 μm. [file Image2.JPEG]

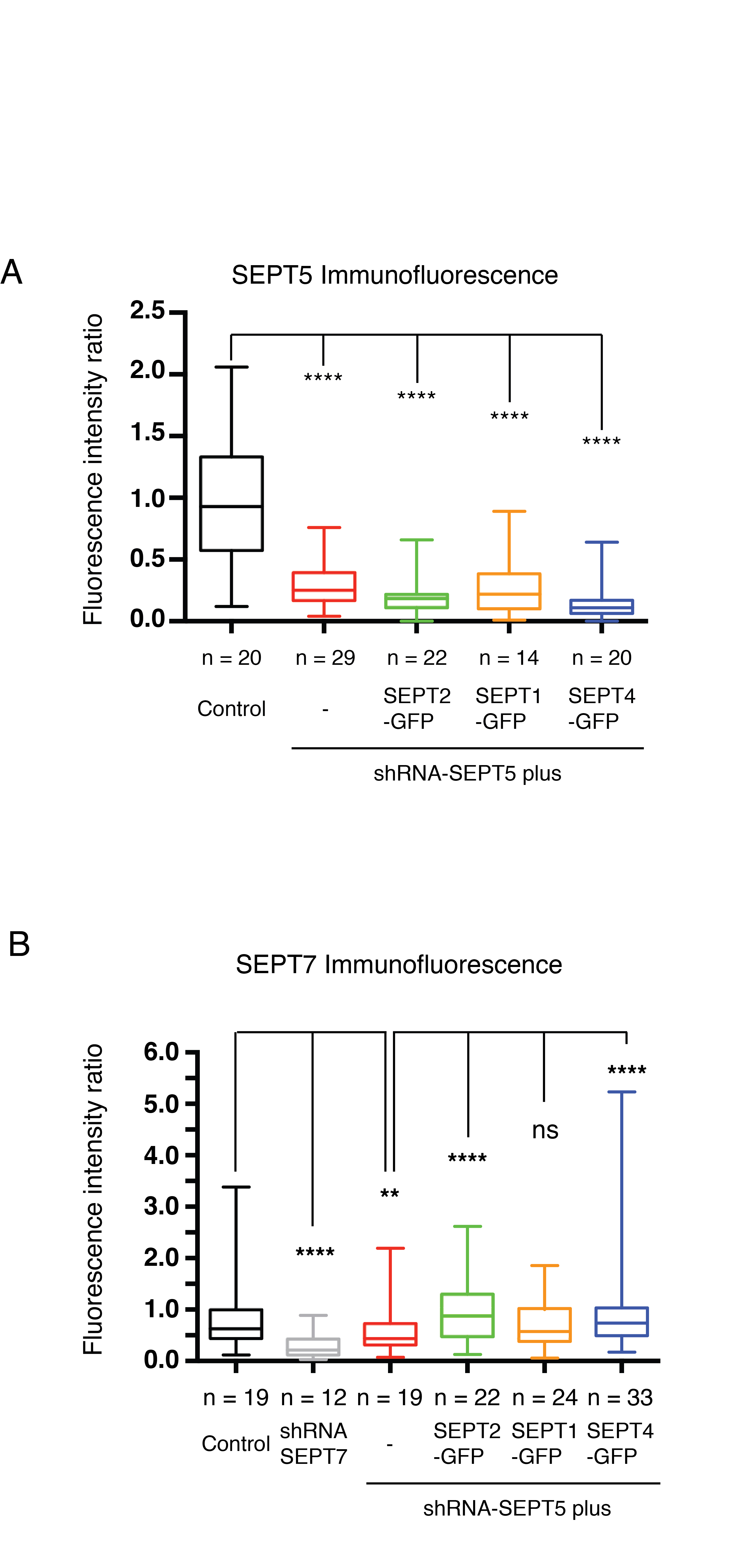

Supplement: Supplementary Figure 3 — Quantification of immunofluorescence data as shown in Supplementary Figures 1, 2. (A) The boxplot illustrates the fluorescence intensity ratios of neurons immunostained against SEPT5. The transfection of the empty pSuper vector served as a negative control, whereas the same vector containing the shRNA against SEPT5 was employed as a positive control. In all neurons cotransfected with shRNA against SEPT5 and SEPTX-GFP, SEPT5 was efficiently reduced. An unpaired Mann-Whitney test was performed to assess significance (****p < 0.0001). “n” stands for the number of cells. (B) The fluorescence intensity ratios of neurons immunostained against SEPT7 illustrate that transfection of shRNA against SEPT7 efficiently downregulates the protein levels of SEPT7 in neurons (****p < 0.0001). In neurons downregulated for SEPT5 the fluorescence intensity ratio of SEPT7 is reduced slightly but significantly (**p = 0.0029). A significant increase of the FI SEPT7 signal in SEPT2-GFP/shRNA SEPT5 expressing neurons and SEPT4GFP/shRNA SEPT5 expressing neurons compared to shRNAS5 was detected (****p < 0.0001). No statistically significant difference was observed for the FI SEPT7 signal from SEPT1-GFP/ shRNA SEPT5 expressing neurons compared to the FI SEPT7 signal from only shRNAS5 expressing neurons (ns, p = 0.1221). An unpaired, two-tailed Mann-Whitney test was performed to assess significance. “n” stands for the number of cells. [file Image3.JPEG]

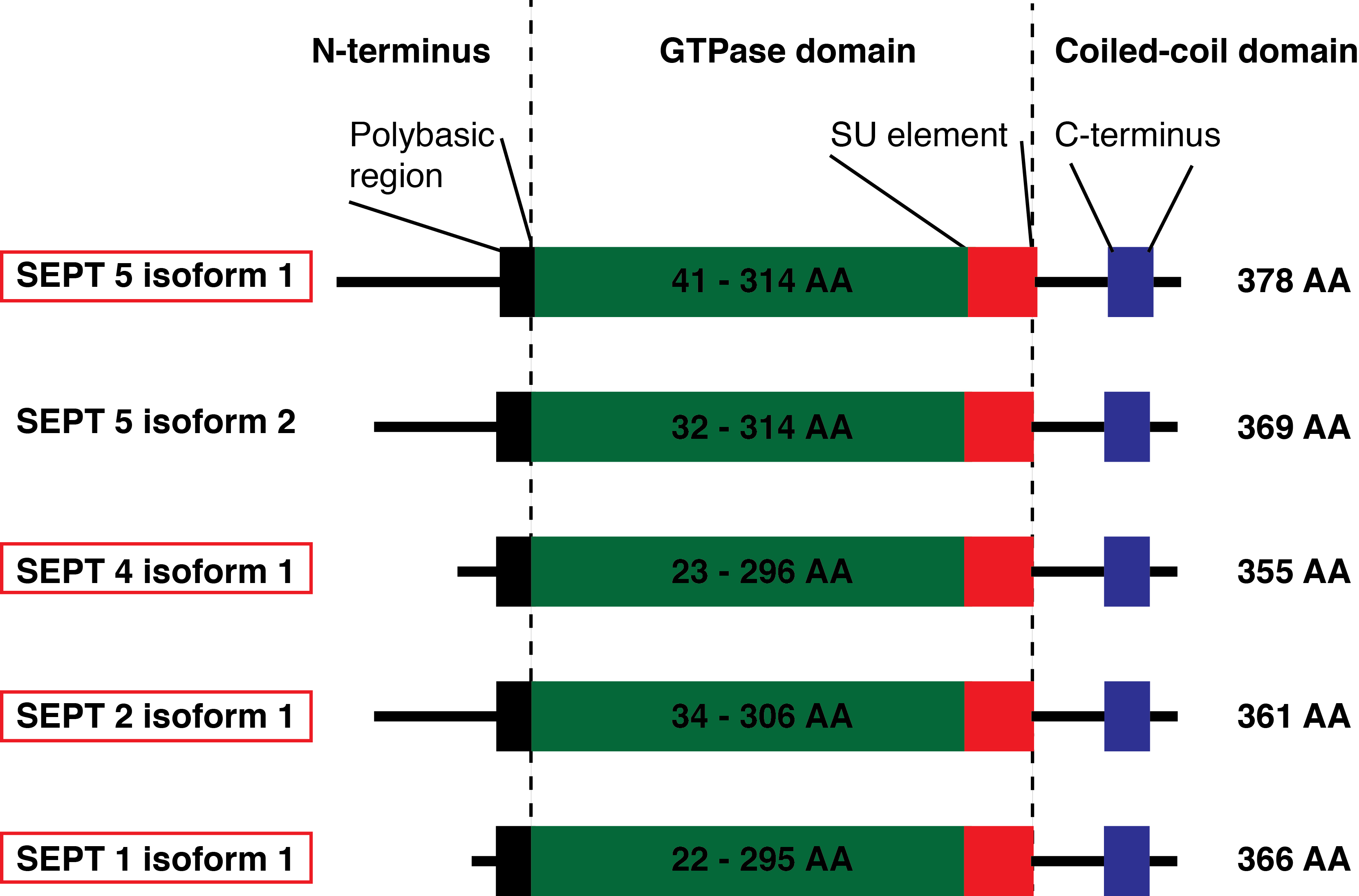

Supplement: Supplementary Figure 4 — Scheme showing SEPT2 group isoforms of Rattus norvegicus. All isoforms listed are known sequences from cDNA cloning. Shown are the common sequence features that the SEPT2 group isoforms share: a black bar describes the N-terminus that is very variable in length between the different SEPT2 group isoforms. The polybasic region is shown by a black box. The green and red boxes comprise the GTPase domain and septin unique element. All isoforms have a coiled-coil domain in the C-terminus in common. Isoforms that were used in this study are highlighted with a red square. [file Image4.JPEG]
