# Supplementary material for: Functional Redundancy of Septin Homologs in Dendritic Branching
Source: Front Cell Dev Biol. 2017 Feb 20;5:11. doi: 10.3389/fcell.2017.00011 (PMC5316521; doi:10.3389/fcell.2017.00011)
Supplement: Supplementary Table 2 — P-values of Sholl analysis. P-values were calculated to compare the control morphology with neurons depleted of SEPT7 or SEPT5 and with neurons depleted of SEPT5 and cotransfected with SEPT1-GFP, SEPT2-GFP, or SEPT4-GFP. P < 0.02 are highlighted in red. [file Table2.docx]

**Supplementary table 2: P-values of Sholl analysis.** P-values were calculated to compare the control morphology with neurons downregulated of SEPT7 or SEPT5 and with neurons downregulated of SEPT5 and cotransfected with SEPT1-GFP, SEPT2-GFP or SEPT4-GFP. P-values < 0.02 are highlighted in red.

| **Control/Distance in μm** | **shRNA SEPT5** | **shRNA SEPT7** | **shRNA SEPT5/ SEPT1-GFP** | **shRNA SEPT5/ SEPT2-GFP** | **shRNA SEPT5/ SEPT4-GFP** |
| --- | --- | --- | --- | --- | --- |
| 20 | 0.8279 | 0.4028 | < 0.0001 | 0.002 | 0.95 |
| 30 | 0.192 | 0.006 | < 0.0001 | 0.076 | 0.79 |
| 40 | 0.018 | 0.036 | < 0.0001 | 0.1124 | 0.47 |
| 50 | 0.0002 | 0.002 | < 0.0001 | 0.6555 | 0.85 |
| 60 | < 0.0001 | 0.001 | < 0.0001 | 0.5577 | 0.23 |
| 70 | < 0.0001 | 0.0002 | < 0.0001 | 0.7353 | 0.096 |
| 80 | < 0.0001 | < 0.0001 | < 0.0001 | 0.8842 | 0.13 |
| 90 | < 0.0001 | < 0.0001 | < 0.0001 | 0.6905 | 0.12 |
| 100 | < 0.0001 | < 0.0001 | < 0.0001 | 0.3904 | 0.05 |
| 110 | < 0.0001 | 0.001 | 0.003 | 0.4602 | 0.04 |
| 120 | < 0.0001 | 0.002 | 0.001 | 0.9304 | 0.018 |
| 130 | < 0.0001 | 0.001 | 0.003 | 0.5312 | 0.008 |
| 140 | < 0.0001 | 0.0016 | 0.01 | 0.3613 | 0.017 |
| 150 | < 0.0001 | 0.05 | 0.02 | 0.9003 | 0.035 |
