# Supplementary material for: Functional Redundancy of Septin Homologs in Dendritic Branching
Source: Front Cell Dev Biol. 2017 Feb 20;5:11. doi: 10.3389/fcell.2017.00011 (PMC5316521; doi:10.3389/fcell.2017.00011)
Supplement: Supplementary Table 3 — SEPT2 group isoforms of Rattus norvegicus. We listed all cloned and predicted isoforms with the Uniprot and NCBI Protein ID reference. We provide the literature reference for the isoforms used in this study in the last column. [file Table3.docx]

**Supplementary table 3: SEPT2 group isoforms of *Rattus norvegicus*.** We listed all cDNA cloned and predicted isoforms with the Uniprot and NCBI Protein ID reference. We provide the literature reference for the isoforms used in this study in the last column.

| **Septin** | **Uniprot** | **NCBI Protein ID** | **Isoform** | **Reference** |
| --- | --- | --- | --- | --- |
| **SEPT 5** | **Q9JJM9-1** | [NP_446383.4](https://www.ncbi.nlm.nih.gov/protein/829569725) | Used in this study | Toda et al., 2000, Biochem and Biophys Research Comm |
|  | **Q9JJM9-2** |  | Isoform 2 |  |
|  | **Q9JJM9-3** |  | Isoform 3 |  |
| **SEPT4** | A0JN02-1 | [AAI26070.1](http://www.ncbi.nlm.nih.gov/protein/116487725) | Used in this study | Strausberg et al., 2002, PNAS |
|  |  | [NP_001011893.1](https://www.ncbi.nlm.nih.gov/protein/58865356) | Septin 4 |  |
|  |  | [XP_017452607.1](https://www.ncbi.nlm.nih.gov/protein/1046848850) | Predicted Isoform – X1 |  |
|  |  | [XP_017452608.1](https://www.ncbi.nlm.nih.gov/protein/1046848852) | Predicted Isoform – X2 |  |
|  |  | [XP_017452609.1](https://www.ncbi.nlm.nih.gov/protein/1046848854) | Predicted Isoform – X3 |  |
|  | A0A096MJN4 | [XP_017452610.1](https://www.ncbi.nlm.nih.gov/protein/1046848856) | Predicted Isoform – X4 |  |
|  |  | [XP_017452611.1](https://www.ncbi.nlm.nih.gov/protein/1046848859) | Predicted Isoform – X5 |  |
|  |  | [XP_017452612.1](https://www.ncbi.nlm.nih.gov/protein/1046848861) | Predicted Isoform – X6 |  |
|  | E9PST0 | [XP_017452613.1](https://www.ncbi.nlm.nih.gov/protein/1046848863) | Predicted Isoform – X7 |  |
|  |  | [XP_017452614.1](https://www.ncbi.nlm.nih.gov/protein/1046848865) | Predicted Isoform – X8 |  |
|  | A0A096MJW0 | [XP_017452615.1](https://www.ncbi.nlm.nih.gov/protein/1046848867) | Predicted Isoform – X9 |  |
|  |  | [XP_017452616.1](https://www.ncbi.nlm.nih.gov/protein/1046848869) | Predicted Isoform – X10 |  |
|  |  | [XP_017452617.1](https://www.ncbi.nlm.nih.gov/protein/1046848871) | Predicted Isoform – X11 |  |
| **SEPT 2** | Q91Y81-1 | NP_476489.1 | Used in this study | Beites et al., 1999, Nat. Neuroscience |
|  |  | [XP_006245574.1](https://www.ncbi.nlm.nih.gov/protein/564369795) | Predicted Isoform – X1 |  |
| **SEPT1** | Q5EB96-1 | NP_001012478.1 | Used in this study | Strausberg et al., 2002, PNAS |
|  |  | [XP_017444483.1](https://www.ncbi.nlm.nih.gov/protein/1046841562) | Predicted Isoform – X1 |  |
