# Supplementary material for: Functional Redundancy of Septin Homologs in Dendritic Branching
Source: Front Cell Dev Biol. 2017 Feb 20;5:11. doi: 10.3389/fcell.2017.00011 (PMC5316521; doi:10.3389/fcell.2017.00011)
Supplement: Supplementary file 8 [file DataSheet1.docx]

**Supplementary Material**

**Supplementary Figure Legends**

**Supplementary Figure 1: Immunofluorescence staining against SEPT5 after treatment of neurons with shRNA against SEPT5.** Immunofluorescence images show example cells transfected with shRNA against SEPT5, mRFP as a marker (shown in white, top row), endogenous SEPT5 detected by immunofluorescence (red, second row), and SEPTX-GFP (green, third row), where X stands for SEPT1, SEPT2 or SEPT4. SEPT2-GFP and SEPT4-GFP signals cluster along the dendrite, whereas SEPT1-GFP localizes homogenously within the cytoplasm (all in green). Scale bars are 2 μm.

**Supplementary Figure 2: Immunofluorescence staining against SEPT7 after treatment of neurons with shRNA against SEPT5.** Immunofluorescence images show example cells transfected with shRNA against SEPT5, mRFP as a marker (shown in white, top row), endogenous SEPT5 detected by immunofluorescence (red, second row), and SEPTX-GFP (green, third row), where X stands for SEPT1, SEPT2 or SEPT4.

Antibody immunostaining against SEPT7 (red) reveals a strong overlap between SEPT2-GFP/ SEPT7 or SEPT4-GFP/ SEPT7 in the merged images. SEPT2-GFP and SEPT4-GFP localize to higher-order structures at the dendritic spine necks, whereas the SEPT1-GFP signal is spread all over the cell (all in green). Scale bars are 2 μm.

**Supplementary Figure 3: Quantification of immunofluorescence data as shown in Supplementary Figures 1 & 2 (A)** The boxplot illustrates the fluorescence intensity ratios of neurons immunostained against SEPT5. The transfection of the empty pSuper vector served as a negative control, whereas the same vector containing the shRNA against SEPT5 was employed as a positive control. In all neurons cotransfected with shRNA against SEPT5 and SEPTX-GFP, SEPT5 was efficiently reduced. An unpaired Mann-Whitney test was performed to assess significance (**** p < 0.0001). ‘n’ stands for the number of cells **(B)** The fluorescence intensity ratios of neurons immunostained against SEPT7 illustrate that transfection of shRNA against SEPT7 efficiently downregulates the protein levels of SEPT7 in neurons (**** p < 0.0001). In neurons downregulated for SEPT5 the fluorescence intensity ratio of SEPT7 is reduced slightly but significantly (** p = 0.0029). A significant increase of the FI SEPT7 signal in SEPT2-GFP/ shRNA SEPT5 expressing neurons and SEPT4GFP/ shRNA SEPT5 expressing neurons compared to shRNAS5 was detected (****p < 0.0001). No statistically significant difference was observed for the FI SEPT7 signal from SEPT1-GFP/ shRNA SEPT5 expressing neurons compared to the FI SEPT7 signal from only shRNAS5 expressing neurons (ns, p = 0.1221). An unpaired, two-tailed Mann-Whitney test was performed to assess significance. ‘n’ stands for the number of cells.

**Supplementary Figure 4: Scheme showing SEPT2 group isoforms of *Rattus* *norvegicus*.** All isoforms listed are known sequences from cDNA cloning. Shown are the common sequence features that the SEPT2 group isoforms share: A black bar describes the N-terminus that is very variable in length between the different SEPT2 group isoforms. The polybasic region is shown by a black box. The green and red boxes comprise the GTPase domain and septin unique element. All isoforms have a coiled-coil domain in the C-terminus in common. Isoforms that were used in this study are highlighted with a red square.
